# Antibacterial Efficacy of Liposomal Formulations Containing Tobramycin and *N*-Acetylcysteine against Tobramycin-Resistant *Escherichia coli*, *Klebsiella pneumoniae*, and *Acinetobacter baumannii*

**DOI:** 10.3390/pharmaceutics14010130

**Published:** 2022-01-05

**Authors:** Reem E. Alarfaj, Manal M. Alkhulaifi, Ahmed J. Al-Fahad, Shokran Aljihani, Alaa Eldeen B. Yassin, Majed F. Alghoribi, Majed A. Halwani

**Affiliations:** 1 Department of Botany and Microbiology, College of Science, King Saud University, Riyadh 11451, Saudi Arabia; reemalarfaj.95@gmail.com (R.E.A.); manalk@ksu.edu.sa (M.M.A.); 2 Infectious Diseases Research Department, King Abdullah International Medical Research Center, King Saud bin Abdulaziz University for Health Sciences, National Guard Health Affairs, Riyadh 11481, Saudi Arabia; 3 National Center for Biotechnology, Life Science & Environment Research Institute, King Abdulaziz City for Science and Technology (KACST), Riyadh 12354, Saudi Arabia; ajlfahad@kacst.edu.sa; 4 Nanomedicine Department, King Abdullah International Medical Research Center, King Saud bin Abdulaziz University for Health Sciences, National Guard Health Affairs, Riyadh 11481, Saudi Arabia; sh.aljihani@gmail.com; 5 Pharmaceutical Sciences Department, College of Pharmacy, King Saud bin Abdulaziz University for Health Sciences, National Guard Health Affairs, Riyadh 11481, Saudi Arabia; yassina@ksau-hs.edu.sa

**Keywords:** antimicrobial resistance, *Escherichia coli*, *Klebsiella pneumoniae*, *Acinetobacter baumannii*, liposomes, tobramycin, *N*-acetylcysteine, multidrug-resistant, resistant genes

## Abstract

The antibacterial activity and biofilm reduction capability of liposome formulations encapsulating tobramycin (TL), and Tobramycin-*N*-acetylcysteine (TNL) were tested against tobramycin-resistant strains of *E. coli*, *K. pneumoniae* and *A. baumannii* in the presence of several resistant genes. All antibacterial activity were assessed against tobramycin-resistant bacterial clinical isolate strains, which were fully characterized by whole-genome sequencing (WGS). All isolates acquired one or more of AMEs genes, efflux pump genes, OMP genes, and biofilm formation genes. TL formulation inhibited the growth of EC_089 and KP_002 isolates from 64 mg/L and 1024 mg/L to 8 mg/L. TNL formulation reduced the MIC of the same isolates to 16 mg/L. TNL formulation was the only effective formulation against all *A. baumannii* strains compared with TL and conventional tobramycin (in the plektonic environment). Biofilm reduction was significantly observed when TL and TNL formulations were used against *E. coli* and *K. pneumoniae* strains. TNL formulation reduced biofilm formation at a low concentration of 16 mg/L compared with TL and conventional tobramycin. In conclusion, TL and TNL formulations particularly need to be tested on animal models, where they may pave the way to considering drug delivery for the treatment of serious infectious diseases.

## 1. Introduction

**Figure 1:**
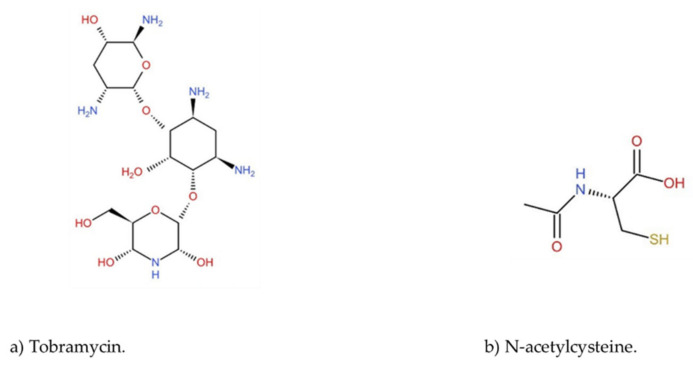
(**a**) Chemical structure of tobramycin; (**b**) chemical structure of *N*-acetylcysteine.

Antimicrobial resistance (AMR) has a substantial impact on human health and is becoming a major global health concern [1,2]. AMR has increased globally both in the community and hospital settings, but mainly in intensive-care units (ICUs) [3]. Among antimicrobial-resistant bacteria, Gram-negative bacteria (GNB) form the most serious threat due to the continuous emergence of resistance to almost every class of antibiotics [1]. Multidrug resistance (MDR) is the consequence of multiple bacterial-resistance mechanisms, such as overexpression of efflux pumps and/or certain outer membrane proteins, as a result of gene mutations that alter the bacterial membrane permeability [4]. Aminoglycosides (AG) remain potent antimicrobial agents with a broad-spectrum activity, and are used to treat severe infections caused by aerobic Gram-negative rods [5,6]. They act by binding to the 30S or 50S ribosomal subunits, leading to mRNA miscoding and protein synthesis inhibition [7,8]. Nevertheless, several bacterial species including *E. coli*, *K. pneumoniae* and *A. baumannii* have developed resistance toward AG’s antibiotics, including tobramycin (Figure 1) [9,10].

Meanwhile, *N*-Acetylcysteine (NAC) (Figure 1), an acylated variant of l-cysteine amino acid, is a known antibiotic adjuvant for treating respiratory infections due to its mucolytic activity [11]. Moreover, several reports indicated that NAC has substantial activity against bacterial biofilms [12,13,14,15]. Moreover, NAC might protect against aminoglycoside toxicity as reported in several research studies [16,17,18,19]. It is well known that all AGs, including tobramycin, are associated with severe nephrotoxicity and ototoxicity [20,21,22].

Liposomes are nano-scale spherical membranous vesicles composed of lipids and/or phospholipids; the key characteristic of these structures is their naturally occurring single or multiple bilayer membranes, which confer protection of the loaded drug [23]. Since the 1970s, liposomes have gained attention for the use as a drug carrier, due to their low toxicity and ability to encapsulate both hydrophobic and hydrophilic compounds [24]. Currently, liposomes are versatile drug carriers in pharmaceutical industries. Using liposomes as delivery systems has many advantages, such as decreasing the side effects of the loaded drug, improving stability and activity, and enhancing the drug concentrations at the site of infection [25,26,27,28,29,30]. Moreover, using liposomes to deliver antibiotics at the site of infection decreases the total administered dosage, which limits the evolution of resistance bacteria [24]. The action of the liposomes is quick enough to kill the bacteria even before it can develop resistance [31,32,33]. As reported, the encapsulation of aminoglycosides into liposomes has improved the therapeutic index of these agents, by increasing the accumulation of the drug in the site of infection [34] and reducing the ototoxicity and nephrotoxicity of the drug [35]. In this study, we investigated the ability of liposomal-encapsulated tobramycin and tobramycin-*N*-Acetylcysteine to overcome resistance in *Escherichia coli*, *Klebsiella pneumoniae*, and *Acinetobacter baumannii*. This work is the first study that has examined the antibacterial activity of tobramycin and *N*-acetylcysteine entrapped in liposomes against antimicrobial-resistant Gram-negative bacteria with a known genomic background.

## 2. Materials and Methods

### 2.1. Sample Collection, Identification, and Susceptibility Tests

Antimicrobial-resistant *E. coli* (*n* = 7), *K. pneumoniae* (*n* = 9), *A. baumannii* (*n* = 5), and *Staphylococcus aureus* ATCC 29213 reference strains were obtained from the Clinical Microbiology Laboratory at the Department of Pathology and Laboratory Medicine, King Abdulaziz Medical City (KAMC), Riyadh. Susceptibility testing was performed using the VITEK 2 compact automated system (Biomerieux, Lyon, France). The minimum inhibitory concentration (MIC) of tobramycin was determined using the micro broth dilution method following EUCAST guidelines [36].

### 2.2. Whole Genome Sequencing and Bioinformatic Analysis

Prior to the genome sequencing, the bacterial DNA was extracted using the MagnaPure compact system (Roche, Basel, Switzerland). DNA library was constructed using Nextera XT Library Prep Kit (Illumina, San Diego, CA, USA). Short-read sequences were generated using the Illumina MiSeq System (Illumina, San Diego, CA, USA) with the 2 × 300 bp paired-end protocol. The antimicrobial resistant genes and virulence factors were identified using ABRicate (version 0.9.8) (Seemann T, Github https://github.com/tseemann/abricate, accessed on 10 November 2021) [37] with the Megares [38], Resfinder [39] and virulence factors database (VFDB) [40]. 

### 2.3. Preparation and Characterization of the Tobramycin Liposomes (TL) and Tobramycin-N-Acetylcysteine Liposomes (TNL) Formulations

The liposome nanovesicles were prepared by the rehydration-rehydration vesicles (DRV) method of Alhariri et al. [41]. Basically, the liposomes were prepared by mixing 1,2-Dimyristoyl-sn-glycero-3-phosphoethanolamine (DMPE), 1,2-Dipalmitoyl-sn-glycero-3-phosphocholine (DPPC), and cholesterol (UFC Biotechnology, Amherst, NY, USA) in the molar ratio 4:2:1 in chloroform. A Rotavapor^®^ R-300 (BÜCHI, Flawil, Switzerland) was used to evaporate the chloroform until a lipid film formed, followed by a stream of nitrogen gas for 5 min, to flush any traces of chloroform. The liposomal vesicles were prepared by dissolving 1 mg tobramycin in PBS, pH 7.4, and 1 mg of tobramycin and 50 mM of *N*-acetylcysteine (NAC) in PBS to form the tobramycin liposome (TL), and the tobramycin-NAC liposome (TNL) formulations. The formulations were homogenized by sonication (Ultrasonic processor UPS 125, Maharashtra, India) for 3 min using cycles of a 10 s run and 2 s pause. The Zetasizer (Malvern, UK) was used to determine the size of the prepared liposomes. The liposomes were washed with PBS by centrifugation for 20 min at maximum speed, and then lyophilized for 48 h in the CHRIST lyophilizer (Osterode am Harz, Germany). The liposomes were reconstructed by gradually adding 10% of the original volume of PBS.

### 2.4. Tobramycin Encapsulation Efficiency (EE%) of the TL and TNL Formulations

A microbiological assay was used to measure the tobramycin encapsulated inside the liposomal formulations. Overnight bacterial cultures of *S. aureus* ATCC 29213 were first adjusted to 0.5 MacFarland and then diluted at 5 × 10^5^ CFU/mL in 250 mL of Mueller-Hinton agar (HIMEDIA) and cooled down to 45 °C. The suspensions were later poured into square culture plates. Triton X-100 (UFC biotechnology, Amherst, NY, USA) (0.2%) was used to lyse the liposomes and release the encapsulated antimicrobial agents for 30 min at 37 °C. After solidification of the seeded plate, 6 mm diameter wells were made and filled with 20 μL of the liposome lysate. The plates were incubated at 37 °C for 24 h, and the average of the inhibition zones was measured. In addition, the quantification of the tobramycin was also performed for the confirmation of the results by using an ultra-high-performance liquid chromatography–tandem mass spectrometer (UHPLC-MS/MS). The UHPLC system consisted of an LPG-300RS quaternary rapid separation pump with an integrated degasser, WPS-300TRS autosampler, TCC-300RS Column compartment and Xcalibur^TM^ 4.3 software (Revision A, Thermo Fisher Scientific, Waltham, MA, USA) [42]. All samples were centrifuged, filtered through 0.22 μm filters and, in some cases, diluted before analysis. Separation was done with a Thermo Scientific^TM^ Syncronis^TM^ C18 column (100 × 2.1 mm, 3 μm particle size). The oven temperature was maintained at 40 °C, and the mobile phase was LC/MS grade, water plus 0.1% formic acid (A) and methanol, plus 0.1% formic acid (B). A linear gradient program was used at a flow rate of 0.300 mL/min: 0.0–2.0 min 2% (B), 2.0–5.0 min from 2% to 98% (B), 5.0–6.0 min from 98% (B), 6.5–9.0 min from 98% to 2% (B), and finally 7.0–10.0 min 2% (B). The identification and quantification of tobramycin was performed on a triple quadrupole mass spectrometer (TSQ Altis, Thermo Fisher Scientific). The mass spectrometer was equipped with an electrospray ionization (ESI) source, which was operated at the following conditions: gas temperature was 300 C, sheath gas 50, aux gas 10, capillary voltage: 3500 V, and argon gas was used for the collision cell. Tobramycin was detected in an ESI positive mode at a retention time Rt = 0.59 min and quantified using selected reaction monitoring (SRM). The transition ions (m/z) associated with tobramycin were 468→163 (22 eV), 468→205 (21 eV) and 468→324 (14 eV). A standard calibration curve (R2 = 0.993) was created using eight different concentrations of tobramycin, ranging from 200 to 1000 ppb.

The following equation was used to calculate the encapsulation efficiency:

(1) $$EE\%=\frac{Concentration\;of\;encapsulated\;drug}{Initial\;concentration\;of\;drug\;}\times 100$$

### 2.5. The Stability of the TL and TNL Formulations in Biological and Storage Conditions

Anonymous patient samples of plasma and sputum were obtained after the routine work was done, and before discarding the samples from the Medical Laboratory, King Abdulaziz Medical City, National Guard Hospital, Riyadh, Saudi Arabia. The sputum samples were diluted 1:10 (*w*/*v*) in PBS before autoclaving. The retention of tobramycin in the prepared liposomes was tested at 37 °C in plasma and sputum, and at 4 °C and 37 °C in PBS. The samples were collected at the time intervals 0, 1, 6, 12, 18 and 24 h, and were harvested and centrifuged at 4 °C at 20,000 rpm. The concentration of tobramycin in the supernatants obtained was checked with the agar well-diffusion method, as described in the encapsulation efficiency (EE%) section.

The following equation was used to calculate the retention of the drugs:

(2) $$Retention\;of\;encapsulated\;drug=\frac{Initial\;concentration-released\;concentration\;}{Initial\;concentration\;}\times 100$$

### 2.6. Antibacterial Activity of TL and TNL Formulations

The minimum inhibitory concentration (MIC) and minimum bactericidal concentration (MBC) of the free form of tobramycin, TL and TNL formulations were tested with the micro broth dilution method. Serial dilution (range 1024–16) of the free tobramycin, and the liposomal formulations prepared in the Mueller-Hinton broth were checked on overnight bacterial cultures that were diluted at 5 × 10^5^ CFU/mL. All samples were seeded on Mueller-Hinton agar for the next day to determine the MBC.

(3) $$Decrease\;\%=\frac{Decrease}{New\;number}\times 100$$

### 2.7. Biofilm Reduction of TL and TNL Formulations

The biofilm reduction assay was performed as described by Paula-Ramos et al. with minor modifications [43]. Fresh bacterial cultures, grown in Mueller-Hinton broth, were adjusted at the 0.5 MacFarland standard, diluted (1:100) into fresh media and then incubated in flat-bottom 96-well plates for 72 h at 37 °C in a shaking incubator at 75 rpm. Planktonic cells were removed by washing twice with sterile dH_2_O. After washing, 100 μL of Mueller-Hinton broth was added to each well and the plates were incubated again for 48 h.

After 72 h of incubation, the planktonic cells were removed by washing twice with water, and the treatments were added as follows. The biofilms were treated with the MIC concentration of each isolate and incubated for 24 h at 37 °C, and 50 mM of NAC and the MIC of tobramycin were tested as well.

The biofilms were stained with 0.1% crystal violet at room temperature for 15 min and washed with sterile distilled water. The plates were dried at room temperature and the biofilms were solubilized though incubation with 99% of ethanol for 15 min. The suspension was transferred to a new plate and the optical density (OD) was measured at 570 nm using a SpectraMax (Molecular Devices, San Jose, CA, USA) plate reader.

The reduction percentage was calculated as follows:

(4) $$Decrease=Original\;number\;\left(OD\right)\;-\;New\;number\;\left(OD\right)$$

The original number was the positive biofilm control (no treatment) and the new number obtained after the treatment.

### 2.8. Statistical Analysis

The results were analyzed with XY tables with Graphpad Prism 9 software, (Version 9.3.0) [44]. ANOVA one-way analysis was used to compare the groups and measure the *p*-value. Note: no significant (*ns*) = *p* > 0.05, significant = * *p* ≤ 0.05, very significant = ** *p* ≤ 0.01, and highly significant = *** *p* ≤ 0.001. All experiments were done in triplicate.

## 3. Results

### 3.1. Susceptibility Profiles of the E. coli, K. pneumoniae, and A. baumannii Clinical Isolates

A total of 21 tobramycin resistant clinical isolates were examined in this study. The isolates included *E. coli* (*n* = 7), *K. pneumoniae* (*n* = 9), and *A. baumannii* (*n* = 5).

The results of the Antimicrobial Susceptibility Testing (AST) using the VITEK 2 compact automated system for *E. coli*, *K. pneumoniae*, and *A. baumannii* are provided in the Supplementary Materials. All the *E. coli* isolates were identified as multidrug-resistant organisms (MDRO) (Table S1 from Supplementary Materials). All the tested *K. pneumoniae* isolates were identified as pan-drug-resistant organisms (PDR), except for isolate KP_086 and KP_095, which were classified as extensively drug-resistant organisms (XDR) (Table S2). Regarding the *A. baumannii* isolates, all the isolates were completely resistant to all the tested agents belonging to the third and fourth cephalosporins, beta-lactamase inhibitors, carbapenems, aminoglycosides and fluoroquinolone classes. All of the *A. baumannii* isolates were identified as XDR organisms (Table S3) [45]. 

### 3.2. Whole Genome Sequencing and Bioinformatic Analysis

The whole genome sequencing analysis detected the presence of multiple outer membrane proteins, efflux pump genes, and genes involved in the formation and development of a biofilm in all the tested organisms. Among the detected OMPs, *ompA* in *E. coli*, *A. baumannii*, and *K. pneumonoiae* isolates, and *omp37* in *K. pneumoniae*. Furthermore, the efflux pump gene *acrD* was detected in all *E. coli* and *K. pneumoniae* isolates, with several other efflux pump genes. On the other hand, the majority of *ade* efflux pump genes were present in *A.baumannii* isolates. Biofilm formation genes were detected as well, including *bap*, *csu*, and PNAG, in *A. baumannii*, and *csg* genes in *E. coli* (Only the major genes are listed in Table 1**,** for more details please refer to Table S4).

Moreover, the presence of the aminoglycoside-modifying enzyme genes (AMEs) was detected in all the tested organisms. One or more of the AMEs, particularly the tobramycin-modifying enzymes, were detected in the isolates including *N*-acetyltransferases (ACC), O-adenyltransferases (ANT), O-phosphotransferases (APH), and methyltransferase *armA* and *rmtF* (for more details please refer to Table S5).

### 3.3. TL and TNL Formulation Characterization

In the current study, we succeeded in formulating liposomes with a size less than bacterium size; the size results illustrated that our prepared TL and TNL formulations were 347.33 ± 62.27 and 229.47 ± 47.57 nm, respectively (Table 2). The polydispersity index (PDI) of our formulations tended to be more heterogenic in size, as the PDI for TL and TNL were 0.85 and 0.68, respectively, representing the diverse particle sizes (Table 2).

The encapsulation efficiency percentage (EE%) of tobramycin inside the TL and TNL formulations were 7.1 and 12.8, respectively. The TNL had a greater encapsulation efficiency of tobramycin and consequently, a higher released concentration, 127.6 mg/L compared with 71 mg/L for TL (Table 3).

### 3.4. The Minimum Inhibitory Concentration (MIC) and Minimum Bactericidal Concentration (MBC) of Tobramycin against the Clinical Bacterial Isolates

The *E. coli*, *K pneumoniae*, and *A. baumannii* isolates were highly resistant to conventional tobramycin with MICs and MBCs ranging between 32–2048 mg/L (Table 4). The MIC of the conventional tobramycin against the *E. coli* isolates of EC_077, EC_089, EC_162, and EC_219 was at 64 mg/L, and able to eradicate these isolates (MBC) at 128 mg/L. Moreover, the EC_057, EC_068, and EC_083 *E. coli* isolates were suppressed and eradicated by conventional tobramycin at 32 mg/L (MIC) and 64 mg/L (MBC).

For the *K. pneumoniae* isolates, the majority of the isolates were extremely resistant to conventional tobramycin. For instance, the growth of isolates KP_002, KP_026, KP_050, KP_057 and KP_95 were inhibited (MIC) at 1024 mg/L and eradicated at 2048 mg/L (MBC). The MIC were 32, 256 and 512 mg/L, for the isolates KP_019, KP_086, and KP_017, the MBC were 64, 512, and 1024 mg/L, respectively. Lastly, all the *A. baumannii* isolates were resistant to the conventional tobramycin at 128 mg/L MIC and 256 mg/L MBC.

### 3.5. TL and TNL Formulations Stability within Biological and Storage Conditions

**Figure 2:**
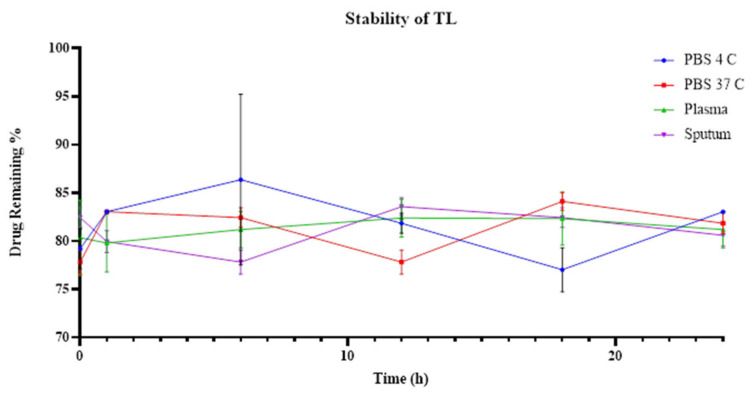
Stability of tobramycin-NAC liposomes in different environments; error bars reflect the SEM.

**Figure 3:**
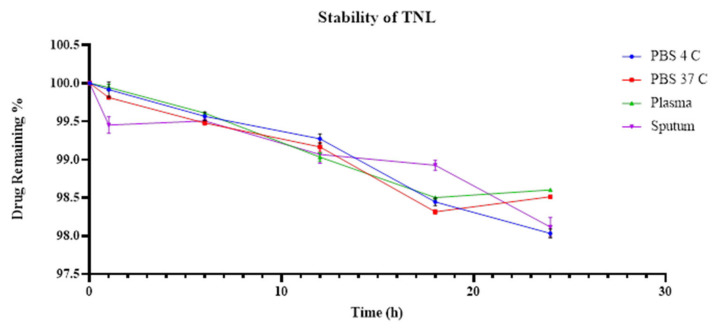
Stability of tobramycin liposomes in different environments; error bars reflect the SEM.

The TNL formulation was highly stable, and time seemed to have little or no effect on the drug release of the liposomes (Figure 2). The TL formulation had a lower stability than our TNL, but maintained a sustained drug release over the 24 h (Figure 3). Of the tested environments, plasma had a constant drug release starting at 79% at hour 1, and 80.7% at hour 24. The same phenomenon was observed with sputum, except for a slight decrease at hour 6 (77.8%). Regarding the stability of TL in PBS at 37 °C and 4 °C, the released drug varied. The highest retention percentage for TL at PBC 4 °C was 81.8% at hour 24, and the lowest was 77.1% at hour 18. For the PBS incubated at 37 °C, the highest retention percentage was 84.18% at hour 18, and the lowest was 77.8% at hour 12. No significant differences were detected in the stability of all the tested conditions for both TL and TNL formulations (*p* value > 0.05).

### 3.6. The Antibacterial Activity of the TL and TNL Formulations against the Genetically Resistant Clinical Bacterial Isolates

The TL and TNL formulations were tested for their minimum inhibitory and bactericidal activity against the multi-drug resistant *E. coli*, *K. pneumoniae*, and *A. baumannii*. The TL and TNL formulations reduced the MIC and MBC against the majority of the isolates (Table 4). The encapsulation of tobramycin inside the TL and TNL formulations improved its activity against seven isolates of *E. coli*. The MIC of these isolates decreased one-fold or two-fold, alternatively, in the cases of EC_057, EC_068, EC_083, EC_162 and EC_219. For EC_089, the MIC significantly decreased ~3-fold. The encapsulation of tobramycin and NAC decreased the MIC of *E. coli*, approximately one-fold against EC_057, EC_068 and SA0219, and two-fold against EC_089 and EC_162. The isolate EC_089 was positive for the detected genes, although it did not harbor any of the tobramycin-modifying enzymes. The MIC of the free tobramycin for this isolate was 64 mg/L, and the encapsulation of tobramycin in the liposomal formulation TL decreased the MIC three-fold (8 mg/L). The co-encapsulation of tobramycin and NAC in a liposomal formulation (TNL) decreased two-fold for this particular isolate (MIC = 16 mg/L).

Overall, the TL had a greater activity against *K. pneumoniae* than *E. coli*, which may be due to the different genetic profile of the two species. The decreased MIC of the TL against *K. pneumoniae* was remarkable. Six isolates were highly resistant to tobramycin (MIC 1024 mg/L) and the MIC decreased six-fold against KP_002, KP_026, KP_050, KP_057, KP_059 and KP_095. The activity was also observed for the TNL.

All the isolates of *A. baumannii* were highly resistant to tobramycin, with a MIC of 128 mg/L. The tested *A. baumannii* isolates were not affected by the TL formulation with no improvement, as the MIC results of the TL formulation were comparable to the conventional tobramycin. The TNL had a great activity against all the *A. baumannii* isolates (MIC = 16 mg/L). This phenomenon could indicate the possible synergy between tobramycin and *N*-acetylcysteine, particularly against *A. baumannii*.

### 3.7. Biofilm Reduction Activity of TL and TNL Formulations against Clinical Bacterial Isolates

In this study, we tested the reduction activity of the liposomal formulations against mature biofilms formed by the selected Gram-negative clinical bacterial isolates with genetical mutations.

**Figure 4:**
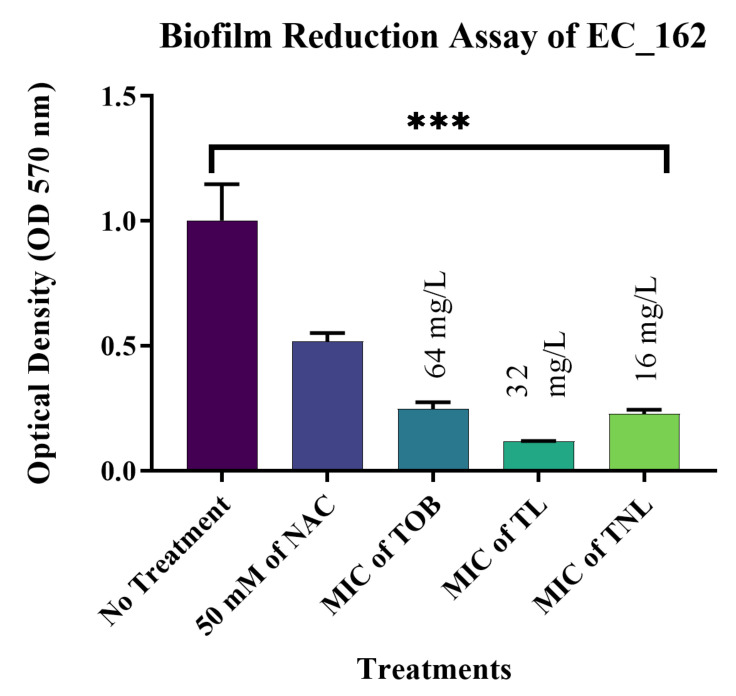
Biofilm reduction assay for isolate EC_162. (*p* value = 0.0003, *** highly significant; error bars reflect the standard error of the mean (SEM)).

**Figure 5:**
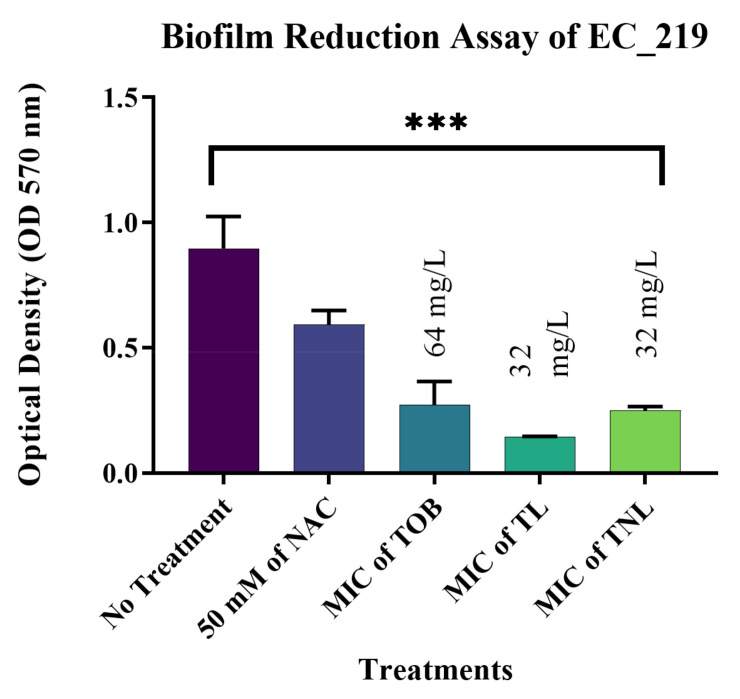
Biofilm reduction assay for isolate EC_219. (*p* value = 0.0008, *** highly significant; error bars reflect the SEM).

**Figure 6:**
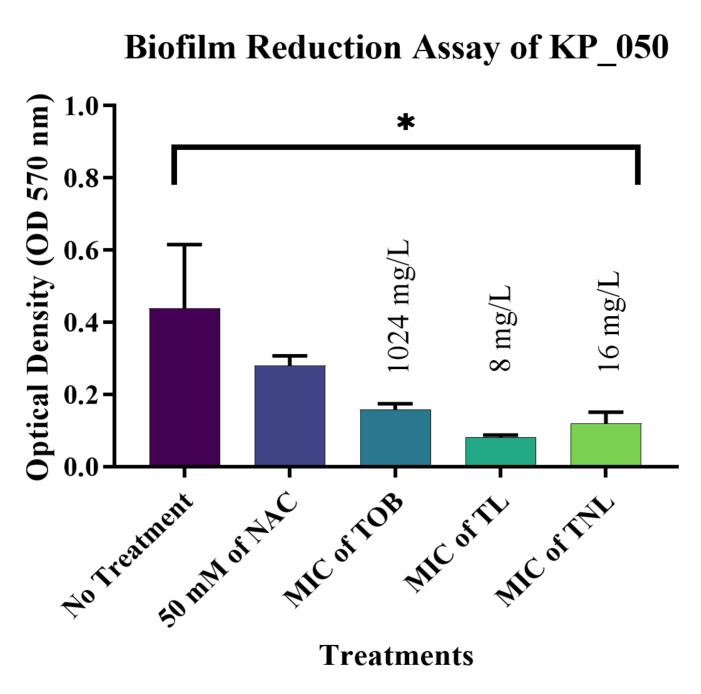
Biofilm reduction assay of isolate KP_050. (*p* value = 0.0343, * significant; error bars reflect the SEM).

**Figure 7:**
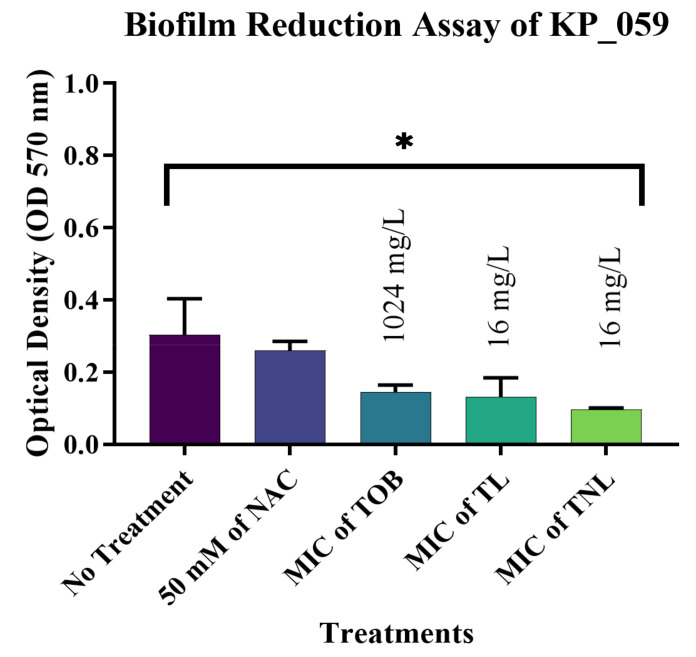
Biofilm reduction assay of isolate KP_059. (*p* value = 0.0411, * significant; error bars reflect the SEM).

**Figure 8:**
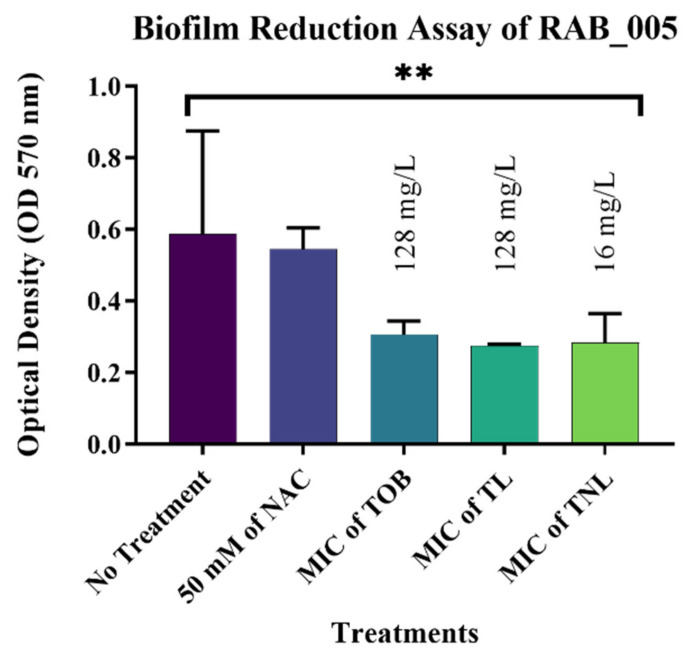
Biofilm reduction assay of isolate RAB_005. (*p* value = 0.0027, ** very significant; error bars reflect the SEM).

**Figure 9:**
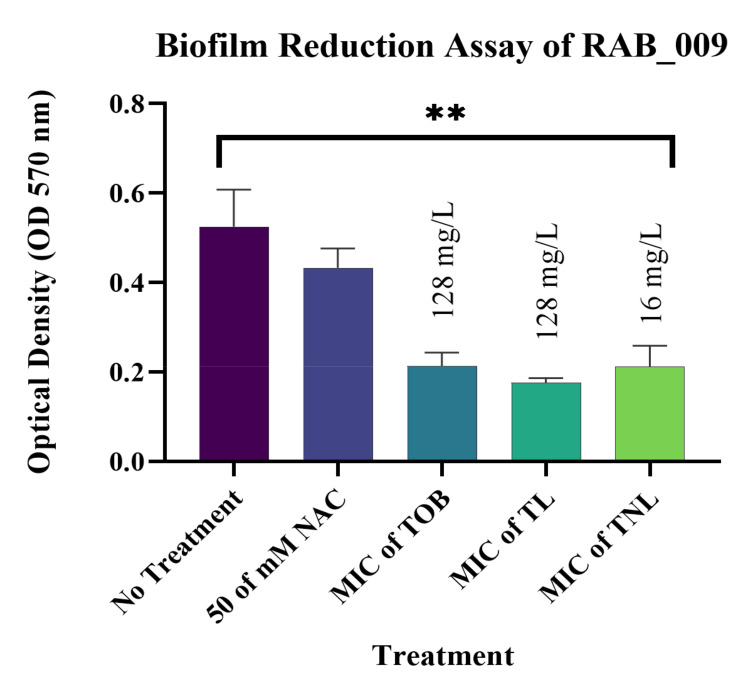
Biofilm reduction assay od isolate RAB_009. (*p* value = 0.0027, ** very significant; error bars reflect the SEM).

The most significant results were observed against biofilms formed by the *E. coli* isolates. The current study confirmed that treatment with 50 mM of the free form of NAC can reduce mature biofilms formed by EC_162 (*p* value = 0.0003) and EC_219 (*p* value = 0.0008) isolates by 48.35% and 33.82%, respectively (Figure 4 and Figure 5). However, encapsulating NAC and tobramycin inside liposomes increased the reduction percentage to 77.18% and 72.04% against the same isolates. The same was observed for biofilms formed by *K. pneumoniae* (Figure 6 and Figure 7). The encapsulation of tobramycin inside liposomal formulations improved its reduction percentage against all of the tested isolates. For *K. pneumoniae* biofilms, the reduction percentage of KP_050 and KP_059, when treated with free NAC, were 36.15% and 14.22%, which increased to 72.71% and 68.20% when treated with TNL, (Figure 6 and Figure 7). The *A. baumannii* strains exhibited a higher sensitivity to the TNL formulation, though the strains remained resistant to conventional tobramycin or encapsulated tobramycin inside a liposomal formulation (TL). Interestingly, the liposomal formulations that contained the TNL were able to maintain the biofilms of *A. baumannii* strains at a very low concentration (16 mg/L), and the other drug forms did the same, but at higher concentration (128 mg/L) (Figure 8 and Figure 9).

## 4. Discussion

*Escherichia coli*, *Klebsiella pneumoniae*, and *Acinetobacter baumannii* are bacterial threats for public health, due to the raised concern of antibiotic resistance [46,47,48,49]. Tobramycin is a family member of aminoglycoside antibiotics, which have been used for treating infections caused by the abovementioned pathogens [50,51,52]. Scientifically, it has been proven that these bacteria are aggressively resistant to antibiotics in biofilm mode [53,54,55]. As documented, several research groups have reported the activity of NAC against bacterial biofilm formation, as well as its antibacterial activities [12,13,14], [56,57,58,59]. Furthermore, it has been reported by numerous research groups that NAC can be encapsulated inside liposomal formulations [60,61,62,63,64,65] Therefore, we considered modifying the conventional tobramycin formula by using drug delivery systems; we are the first research group to have developed tobramycin with NAC inside the liposomal formulation. We have reported here the superior activity of our liposomal NAC formulation (TNL), as an antibacterial formula that has the mucolytic ability to reduce bacterial biofilm formations and reduce tobramycin resistance.

In this study, we used the multidrug-resistant organisms (MDRO) of *E. coli* isolates, the pan-drug resistant organisms (PDR) of *K. pneumoniae* isolates, and drug-resistant organisms (XDR) of *A. baumannii* isolates. The results shown in the Supplementary Materials sheets show that the TL and TNL formulations were able to reduce bacterial resistance phenomenally, as shown in Table 3. We investigated the genetic variations of these particular bacteria, in order to explain the antibacterial activity of our liposomal NAC formulations. We found that the outer membrane proteins (OMPs), including *ompA* and the efflux pump *acrABD-tolC*, were present in all seven *E. coli* isolates. Notably, the AcrAD-TolC and KDPE, and MTD efflux pumps were also present in all tested *E. coli* isolates. These pumps are responsible for the resistance to multiple aminoglycosides agents, including tobramycin. The deletion of the transporter gene (*arcD*) results in a reduction of MIC for the aminoglycoside agents, including tobramycin, gentamycin, amikacin, kanamycin, and neomycin [66,67]. The whole-genome data revealed that all the tested *E. coli* isolates were positive for the presence of *acrD*, and all were highly resistant to tobramycin. The operon genes, including *csgA*, *csgB*, *csgD*, *csgF*, and *csgG*, were also detected in all the *E. coli* clinical isolates, and were involved in the formation and development of a biofilm. Curli fimbriae (*csgA*) are surface protein genes that are essential for many functions, including adhesion, cell aggregation, and biofilm formation [68]. In addition, mutations of the *csgA* gene can cause a defect in the bacterial surface attachment ability and biofilm formation [69]. (Table S4). In addition, all *K. pneumoniae* clinical isolates expressed different outer membrane protein (OMP) genes, such as *omp37* and *ompA*. Many multidrug efflux pump genes were also detected, including *acrA*, *acrB*, *acrD*, *mdt*, and *emrD*. (Table S4).

With regard to the *A. baumannii* isolates, genetic data revealed that they already contained all the genes listed above, the same as *E. coli* and *K. pneumoniae*, except for one efflux pump system. (Table S4). Interestingly, we observed that the outer membrane A genes (*ompA*), which have a major role in the adherence, invasion, and biofilm formation of *A. baumannii*, were present in all the isolates. Moreover, we observed the presence of two of the three efflux pump systems in all the isolates, namely *adeABC* and *adeFGH*. The AdeABC genes, *adeA*, *adeB* and *adeC*, were present in all the *A. baumannii* isolates, and all were associated with a high level of resistance to tobramycin. As mentioned in the Table S4, the overexpression of these pumps plays a role in biofilm formation in the clinical isolates of *A. baumannii* [70].

Furthermore, the presence of the aminoglycoside-modifying enzyme genes (AMEs) were also detected in the *E. coli*, *K. pneumoniae*, and *A. baumannii*. In the *E. coli* (Table S5), one or more of the AMEs, particularly the tobramycin-modifying enzymes, were detected in the isolates. However, in the isolate EC_089, none of the tobramycin modifying enzymes were detected, even though this particular strain displayed remarkable resistance to tobramycin, recorded at 64 and 128 mg/L for MIC and MBC, respectively. Notably, we detected other aminoglycoside-modifying enzymes for this isolate (EC_089), including *N*-acetyltransferases (ACC) *aac(3)-IIa* and *aac(6′)-Ib-cr*, O-adenyltransferases (ANT), such as *ant(2″)-Ia*, and O-phosphotransferases (APH) which included *aph(6)-Id* and *aph(3″)-Ib*. Similarly, the same occurred in the *K. pneumoniae* isolates, and all the isolates were encoded with more than one of the AME genes. The isolates KP_002, KP_019, KP_045, KP_050, KP_057, KP_059, and KP_095 were encoded with the aminoglycoside resistant methyltransferase (*armA*) gene, except for one isolate, KP_017, which harbored the *rmtF* gene. (Table S5). Likewise, *A. baumannii* isolates were not different from the *E. coli* and *K. pneumoniae* isolates. All the isolates harbored the *aph(6)-Id*, *aph(3″)-Ib*, and *armA* (Table S5).

Finally, in terms of the biofilm formation genes, we detected several genes including the biofilm-associated protein (*bap*), Csu fimbriae (*csuA*, *csuA/B*, *csuB*, *csuC*, *csuD*, *csuE*), and PNAG (*pgaA*, *pgaB*, *pgaC*, *pgaD*). In addition, the *bfmRS* two-component system and *abaIR* quorum-sensing system were present in all three bacterial isolates.

On other hand, the sizes of the liposome formulations were expected to range from 25 nm to 2.5 μm, based on which they were classified as small (≤100 nm), intermediate (100–250 nm), large (≥250 nm) or giant (>1 μm) [71,72] The average size of bacterial cells is approximately 1 μm, and for liposomal formulations to fuse properly with the bacterial membrane and release their contents, they must be smaller than the bacterial cells. The size of the nanoparticle is an important factor in drug delivery to eukaryotic cells. It contributes to the tissue distribution, pharmacokinetics, and clearance of these delivery systems. The size of the nanocarriers differs based on the route of administration, for example, for intravenous administration, the particle size can be ranged between 200 nm and 2000 nm [73,74].

As reported by Messiaen et al., 2013, the size of the prepared tobramycin liposomes were 426.3 (±26.4) and 228.5 (±34.9), making the sizes of the TL and TNL average [71]. However, the size of the TNL liposomes were closer to those described by Hasanin and others, which had a mean diameter of 200 nm [72,75]. The polydispersity index (PDI), also known as the heterogeneity index, is a description of the size distribution in the tested sample. The PDI values range from 0.0 (homogeneity) and 1.0 (heterogeneity) [76,77]. Our encapsulation concentration was close to the previously reported DPPC/cholesterol-tobramycin liposomes by Messiaen et al., 2013. Their encapsulation was 141 (±35) ug/mL, and higher than the liposomes reported by Halwani et al., 2008 (0.2 mg/mL) [30,71]. The stability tests of the TL and TNL formulations supported a better understanding of the drug release in different biological and storage environments. Though the stability tests of our formulation gave us a better understanding of the drug release in different biological and storage environments, the stability of the TL formulation can be improved by implementing different preparation methods or changing the physical and chemical conditions of the current preparation method, such as heat and pH levels, to increase the entrapment of the drugs [78].

Overall, MIC and MBC of the TL and TNL were significantly decreased compared with the conventional tobramycin, which indicates that we succeeded in enhancing the antibiotic activity by using liposomes as a drug vehicle. Prior studies have also succeeded in improving the delivery of conventional tobramycin [28,76]; for instance, Marier et al., 2003, used liposomal tobramycin to treat pulmonary infections caused by *Pseudomonas aeruginosa* in rats [75]. Moreover, tobramycin liposomes exhibited strong bactericidal activity against a large range of resistant bacteria, including Gram negative bacteria [79].

This could be due to the presence of the *armA* gene (the aminoglycoside resistant methyltransferase), in all the *A. baumannii* isolates, which confers a high level of resistance to a wide range of aminoglycoside agents, including tobramycin [80,81,82]. This gene functions at the target site through methylation, which prevents the drug from recognizing its target site [80]. Biofilms are a community of bacteria embedded in a self-produced extracellular polymeric substance. The complex structure of biofilms prevents the entry of most antibiotics, and can mediate the adhesion of bacteria to various surfaces. Bacteria within biofilm can be up to 1000 times more resistant than its planktonic phenotypes [83]. It is known that NAC has antibiofilm activity. The activity includes several mechanisms, including the reduction of the biofilm formation process, the reduction of the matrix production or the disruption of the formed biofilms as in the current study [12,58,84,85]. Scientifically, the *N*-acetyl cysteine activity may have played a role as an antibiofilm and mucolytic agent [11,12,19]. The increased reduction activity of the encapsulated tobramycin compared with free tobramycin was reported by Sans-Serramitjana et al., (2017), who reported that the encapsulation resulted in a decreased minimal biofilm eradication concentration (MBEC) of the used drug for all tested *P. aeruginosa* isolates [86]. These results are compatible with our results; the encapsulation of tobramycin increased the reduction activity against the tested organisms. The activity of tobramycin in the liposome forms were also reported against *Burkhoderia cepacia* biofilms [71]. A study by Marchese et al., was conducted to test the antibiofilm activity of NAC alone, or with antibiotics on biofilms formed by *E. coli* isolates. The results were similar to the current study [85]. In addition to the NAC inhibition of the biofilm matrix production in all the tested *E. coli* isolates, they found that NAC at concentrations between 2 and 8 mg/mL disrupted mature biofilms. The highest reduction % against *E. coli* was 60% [85].

## 5. Conclusions

The encapsulation of tobramycin and *N*-acetylcysteine successfully reduced the MIC of the resistant high-risk Gram-negative pathogens. In comparison with the conventional form of tobramycin, the encapsulated tobramycin in liposomal (TL) and *N*-acetylcysteine-liposomal (TNL) formulations increased the antibacterial activity against the tested pathogens. The TL and TNL formulations reduced the biomass of the biofilms. Using liposomes as delivery systems may enhance the treatment of infections caused by multidrug-resistant high-risk pathogens. In addition, the encapsulation efficiency and the stability of the prepared formulations can be improved by assessing other preparation methods.

## Figures and Tables

**Table 1:** Remarkable detected genes: outer membrane genes, efflux pump genes and biofilm formation genes in the tested organisms, (Table S4).

Category	*Escherichia coli*	*Acinetobacter baumannii*	*Klebsiella pneumoniae*
Outer membrane proteins (OMPs)	*ompA*	*ompA*	*ompA*, *omp37*
Efflux pumps	*acrA*, *acrB*, *acrD*, *acre*, *acrF*, *acrS*, *mdtA-C*, *mdtE-K*, *mdtM-P*	*adeA-C*, *adeI*, *adeK*, *adeL*, *adeR*, *adeT1*, *adeT2*, *abeM*, *abeS*	*acrA*, *acrB*, *acrD*
Biofilm formation	*csgB*, *csgD*, *csgF*, *csgG*	*pgaA-D*, *csuA-D*, *bap*, *csgB*, *csgD*, *csgF*, *csgG*	-

**Table 2:** The average of triplicate reads of the particles size and PDI.

Formula	Peak	PDI
TL	347.33 ± 62.27	0.85
TNL	229.47 ± 47.57	0.68

**Table 3:** The Encapsulation efficiencies EE% and the released concentrations of the prepared formulations.

Liposomal Formulations	Entrapped Concentration (mg/L)	EE%
TL	71	7.1
TNL	127.6	12.8

TL: tobramycin liposomes. TNL: tobramycin-*N*-acetylcysteine liposomes.

**Table 4:** The MIC and MBC of tobramycin, TL, and TNL against *E. coli*, *K. pneumoniae*, and *A. baumannii*.

Isolate ID	Tobramycin		TL		TNL	
	(mg/L)					
	MIC	MBC	MIC	MBC	MIC	MBC
*E. coli* clinical isolates						
EC_057	32	64	32	64	16	32
EC_068	32	64	32	64	16	32
EC_077	64	128	32	64	16	32
EC_083	32	64	32	64	16	32
EC_089	64	128	8	16	16	32
EC_162	64	128	32	64	16	32
EC_219	64	128	32	64	32	64
*K. pneumoniae* clinical isolates						
KP_002	1024	2048	8	16	16	32
KP_017	512	1024	32	64	32	64
KP_019	32	64	16	32	32	128
KP_026	1024	2048	8	16	16	32
KP_050	1024	2048	8	16	16	32
KP_057	1024	2048	16	32	16	32
KP_059	1024	2048	16	32	16	32
KP_086	256	512	16	32	32	64
KP_095	1024	2048	16	32	32	64
*A. baumannii* clinical isolates						
RAB_005	128	256	128	256	16	32
RAB_009	128	256	128	256	16	32
RAB_014	128	256	128	256	16	32
RAB_030	128	256	128	256	16	32
RAB_055	128	256	128	256	16	32
*S. aureus* ATCC 29213 *	4	8				

* This was done for validation purposes.

## Data Availability

Data are contained within the article and Supplementary Materials.

## Supplementary Materials

The following supporting information can be downloaded at: https://www.mdpi.com/article/10.3390/pharmaceutics14010130/s1. Table S1. MIC of *E. coli* isolates by VITEK. Table S2. MIC of *K. pneumoniae* isolates by VITEK. Table S3. MIC of *A. baumannii* isolates by VITEK. Table S4. Detected efflux pumps, outer membrane, and biofilm formation genes in *E. coli*, *K. pneumoniae* and *A. baumannii*. Table S5. Detected Aminoglycosides modifying enzymes, 16S rRNA methyltransferases, and other genes in *E. coli*, *K. pneumoniae* and *A. baumannii*.
