# Supplementary material for: Antibacterial Efficacy of Liposomal Formulations Containing Tobramycin and N-Acetylcysteine against Tobramycin-Resistant Escherichia coli, Klebsiella pneumoniae, and Acinetobacter baumannii
Source: Pharmaceutics. 2022 Jan 5;14(1):130. doi: 10.3390/pharmaceutics14010130 (PMC8778299; doi:10.3390/pharmaceutics14010130)
Supplement: Supplementary file 1 [file pharmaceutics-14-00130-s001.zip › pharmaceutics-1487499-supplementary.pdf]

pharmaceutics

MDPI

Supplementary Material

Antibacterial Efficacy of Liposomal Formulations Containing Tobramycin and *N*-acetylcysteine against Tobramycin-Resistant *Escherichia coli*, *Klebsiella pneumoniae*, and *Acinetobacter baumannii*

Reem E. Alarfaj [1,2], Manal M. Alkhulaifi [1], Ahmed J. Al-Fahad [3], Shokran Aljihani [4], Alaa Eldeen B. Yassin [5], Majed F. Alghoribi [2,*] and Majed A. Halwani [4,*]

[1] Department of Botany and Microbiology, College of Science, King Saud University, Riyadh 11451, Saudi Arabia; reemalarfaj.95@gmail.com (R.E.A.); manalk@ksu.edu.sa (M.M.A.)
[2] Infectious Diseases Research Department, King Abdullah International Medical Research Center, King Saud bin Abdulaziz University for Health Sciences, National Guard Health Affairs, Riyadh 11481, Saudi Arabia
[3] National Center for Biotechnology, Life Science & Environment Research Institute, King Abdulaziz City for Science and Technology (KACST), Riyadh 12354, Saudi Arabia; ajlfahad@kacst.edu.sa
[4] Nanomedicine Department, King Abdullah International Medical Research Center, King Saud bin Abdulaziz University for Health Sciences, National Guard Health Affairs, Riyadh 11481, Saudi Arabia; sh.aljihani@gmail.com (S.A)
[5] Pharmaceutical Sciences Department, College of Pharmacy, King Saud bin Abdulaziz University for Health Sciences, National Guard Health Affairs, Riyadh 11481, Saudi Arabia; yassina@ksau-hs.edu.sa
* Correspondence: halawanima@ngha.med.sa (M.A.H.); alghoribima@ngha.med.sa (M.F.A.); Tel.: +96-61-1429-4433 (M.A.H.)

Table S1. MIC of *E. coli* isolates by VITEK.

Isolate ID	EC_057	EC_068	EC_077	EC_083	EC_089	EC_162	EC_219
Specimen type	Urine	Urine	Urine	Urine	Urine	Urine	Urine
SAM	>=32	>=32	>=32	>=32	>=32	>=32	>=32
	R	R	R	R	R	R	R
TIM	>=128	64	64	>=128	>=128	>=128	>=128
	R	I	I	R	R	R	R
TZP	8	<=4	8	8	8	8	>=128
	S	S	S	S	S	S	R
CAZ	>=64	16	16	<=1	4	16	16
	R	R	R	R	R	R	R
FEP	>=64	4	4	<=1	2	8	>=64
	R	R	R	R	R	R	R
ATM	>=64	16	16	<=1	16	>=64	16
	R	R	R	R	R	R	R
ETP	<=0.5	<=0.5	<=0.5	<=0.5	<=0.5	<=0.5	<=0.5
	S	S	S	S	S	S	S
IMP	<=0.25	<=0.25	<=0.25	<=0.25	<=0.25	<=0.25	<=0.25
	S	S	S	S	S	S	S
MEM	<=0.25	<=0.25	<=0.25	<=0.25	<=0.25	<=0.25	<=0.25
	S	S	S	S	S	S	S
AMK	<=2	4	4	8	4	8	8
	S	S	S	I	S	I	I
GEN	>=16	>=16	>=16	4	4	>=16	<=1
	R	R	R	R	R	R	S

TOB	8	>=16	>=16	>=16	>=16	>=16	>=16
	R	R	R	R	R	R	R
CIP	>=4	>=4	>=4	>=4	>=4	1	>=4
	R	R	R	R	R	S	R
LVX	>=8	>=8	>=8	>=8	>=8	1	>=8
	R	R	R	R	R	S	R
MIN	4	2	2	<=1	<=1	<=1	>=16
	S	S	S	S	S	S	R
TGC	<=0.5	<=0.5	<=0.5	<=0.5	<=0.5	<=0.5	<=0.5
	S	S	S	S	S	S	S
SXT	>=320	>=320	>=320	>=320	>=320	>=320	>=320
	R	R	R	R	R	R	R

S: Sensitive, R: Resistant, I: Intermediate.

Table S2. MIC of *K. pneumoniae* isolates by VITEK.

**Isolate ID**	**KP_002**	**KP_017**	**KP_019**	**KP_026**	**KP_050**	**KP_057**	**KP_059**	**KP_086**	**KP_095**
Specimen	Fluid	Wound	Urine	Rectal	Blood	Urine	Blood	Sputum	Wound
AMP	R	R	R	R	R	R	R	R	R
	>=32	>=32	>=32	>=32	>=32	>=32	>=32	>=32	>=32
AMC	R	R	R	R	R	R	R	R	R
	>=32	>=32	>=32	>=32	>=32	>=32	>=32	>=32	>=32
PIP/TAZ	R	R	R	R	R	R	R	R	R
	>=128	>=128	>=128	>=128	>=128	>=128	>=128	>=128	>=128
CF	R	R	R	R	R	R	R	R	R
	>=64	>=64	>=64	>=64	>=64	>=64	>=64	>=64	>=64
FOX	R	R	R	R	R	R	R	R	R
	>=64	>=64	>=64	>=64	>=64	>=64	>=64	16	>=64
CAZ	R	R	R	R	R	R	R	R	R
	>=64	>=64	>=64	>=64	>=64	>=64	>=64	>=64	>=64
CRO	R	R	R	R	R	R	R	R	R
	>=64	>=64	>=64	>=64	>=64	>=64	>=64	>=64	>=64
FEP	R	R	R	R	R	R	R	R	R
	>=64	>=64	>=64	>=64	>=64	>=64	>=64	>=64	>=64
IMP	R	I	R	R	I	R	R	R	R
	>=16	2	>=16	>=16	8	>=16	>=16	>=16	>=16
MEM	R	R	R	R	R	R	R	R	R
	>=16	>=16	>=16	>=16	>=16	>=16	>=16	8	>=16
AMK	R	R	R	I	R	R	R	R	R
	>=64	>=64	>=64	32	>=64	>=64	>=64	>=64	>=64
GEN	I	R	I	R	R	R	R	S	I
	4	>=16	4	>=16	>=16	>=16	>=16	<=1	4
CIP	R	R	R	R	R	R	R	R	R
	>=4	>=4	>=4	>=4	>=4	>=4	>=4	>=4	>=4
TGC	R	R	R	R	R	R	R	R	S
	>=8	4	>=8	8	>=8	>=8	>=8	4	<=0.5
NIT	R	R	R	R	R	R	R	R	R
	128	256	256	256	256	256	128	128	128
TMP	R	R	R	R	R	R	R	R	R
TMP-SMX	R	R	R	R	R	R	R	R	R

	>=320	>=320	>=320	>=320	>=320	>=320	>=320	>=320	>=320

S: Sensitive, R: Resistant, I: Intermediate.

Table S3. MIC of *A. baumannii* isolates by VITEK.

**Isolate ID**	**RAB_005**	**RAB_009**	**RAB_014**	**RAB_030**	**RAB_055**
Specimen	Respiratory	Respiratory	Respiratory	Urine	Respiratory
SAM	R	R	R	R	R
	>=32	16	>=32	16	>=32
TIM	R	R	R	R	R
	>=128	>=128	>=128	>=128	>=128
TZP	R	R	R	R	R
	>=128	>=128	>=128	>=128	>=128
CAZ	R	R	R	R	R
	>=64	>=64	>=64	>=64	>=64
FEP	R	R	R	R	R
	>=64	>=64	>=64	>=64	>=64
IMP	R	R	R	R	R
	>=16	>=16	>=16	>=16	>=16
MEM	R	R	R	R	R
	>=16	>=16	>=16	>=16	>=16
GEN	R	R	R	R	R
	>=16	>=16	>=16	>=16	>=16
TOB	R	R	R	R	R
	>=16	>=16	>=16	>=16	>=16
CIP	R	R	R	R	R
	>=4	>=4	>=4	>=4	>=4
LVX	R	R	R	R	R
	>=8	4	>=8	>=8	>=8
MIN	I	S	I	I	I
	8	4	>=16	>=16	>=16
TGC	2	2	4	>=8	4
	S	S	I	R	I
SXT	<=20	<=20	>=320	>=320	<=20
	S	S	R	R	S
	2	>=16	2	<=0.5	<=0.5
	S	R	S	S	S

S: Sensitive, R: Resistant, I: Intermediate.

Table S4. Detected efflux pumps, outer membrane, and biofilm formation genes in *E. coli, K. pneumoniae,*and *A. baumannii.*

Category	Genes	*Escherichia coli*							*Klebsiella Pneumoniae*									*Acinetobacter baumannii*				
		EC_057	EC_068	EC_077	EC_083	EC_089	EC_162	EC_219	KP_002	KP_017	KP_019	KP_026	KP_050	KP_057	KP_059	KP_086	KP_095	RAB_005	RAB_009	RAB_014	RAB_030	RAB_055
Efflux pumpS (EPS)	*abeM*																	■	■	■	■	■
	abeS																	■	■	■	■	■
	acrA	■	■	■	■	■	■	■	■	■	■	■	■	■	■	■	■					
	acrB	■	■	■	■	■	■	■	■	■	■	■	■	■	■	■	■					
	acrD	■	■	■	■	■	■	■	■	■	■	■	■	■	■	■	■					
	acrE	■	■	■	■	■	■	■														
	acrF	■	■	■	■	■	■	■														
	acrS	■	■	■	■	■	■	■														
	adeA																	■	■	■	■	■
	adeB																	■	■	■	■	■
	adeC																	■	■	■	■	■
	adeI																	■	■	■	■	■
	adeK																	■	■	■	■	■
	adeL																	■	■	■	■	■
	adeN																	■	■		■	
	adeR																	■	■	■	■	■
	adeT1																	■	■	■	■	■
	adeT2																	■	■	■	■	■
	amvA																	■	■	■	■	■
	asmA									■	■	■	■	■	■	■	■					
	baer								■	■	■	■	■	■	■	■	■					
	cpxar								■	■	■	■	■	■	■	■	■					
	crp								■	■	■	■	■	■	■	■	■					
	emrB								■	■	■	■	■	■	■	■	■					
	emrD								■	■	■	■	■	■	■	■	■					
	emrR								■	■	■	■	■	■	■	■	■					
	hns									■	■	■	■	■	■	■	■					
	kdeA								■	■	■	■	■	■	■	■	■					
	kdpE	■	■	■	■	■	■	■														
	kmrA								■	■	■	■	■	■	■	■	■					
	kpn								■	■	■	■	■	■	■	■	■					
	kpnE								■	■	■	■	■	■	■	■	■					
	kpnF								■	■	■	■	■	■	■	■	■					
	kpnO								■	■	■	■	■	■	■	■	■					

	marA
	mdtA
	mdtB
	mdtC
	mdtE
	mdtF
	mdtG
	mdtH
	mdtI
	mdtJ
	mdtK
	mdtM
	mdtN
	mdtO
	mdtP
	mexT
	msbA
	oqxA
	oqxB
	phoR
	ramA
	soxS
Outer membrane proteins (OMPs)	*omp37*
	ompA
Biofilm formation	*pgaA*
	pgaB
	pgaC
	pgaD
	csuA/B
	csuB

	csuC																	■	■	■	■	■	■
	csuD																	■	■	■	■	■	■
	csuD																	■	■	■	■	■	■
	bap																	■	■	■	■	■	■
	bfmR																	■	■	■	■	■	■
	bfmS																	■	■	■	■	■	■
	adeh																	■	■	■	■	■	■
	adeg																	■	■	■	■	■	■
	adef																	■	■	■	■	■	■
	csgB	■	■	■	■		■	■															
	csgD	■	■	■	■	■	■	■															
	csgF	■	■	■	■	■	■	■															
	csgG	■	■	■	■	■	■	■															

The black highlight (■) indicates the presence of the gene.

Table S5. Detected Aminoglycosides modifying enzymes, 16S rRNA methyltransferases, and other genes in *E. coli, K. pneumoniae* and *A.baumannii.*

Species	Isolate	Aminoglycosides modyfing enzymes (AMEs)													16S rRNA methyl-transferases		Miscellane-ous		
		N-acetyltransferases					O-nucleotidyl-transferases		Aminoglycoside_O-phosphotransferases										
		aac3	*aac(3)-IIa*	*aac(6′)-Ib3*	*aac(6′)-Ib-Hangzhou*	*aac(6′)-Ib-cr*	*ant(2′′)-Ia*	*ant3-dprime*	*aph(3′)-Ia*	*aph3-prime*	*aph(3′)-VI*	*aph(3′)-VIb*	*aph(6)-Id*	*aph(3′′)-Ib*	*armA*	*rmtF*	*aadA1*	*aadA2*	*aadA5*
Escherichia coli	EC_057	■	■			■		■											■
	EC_068	■	■			■		■											■
	EC_077	■	■			■		■											■
	EC_083					■	■										■		
	EC_089							■					■	■					
	EC_162	■	■			■		■											■
	EC_219					■													
Klebsiella Pneumoniae	KP_002					■		■		■	■		■	■	■		■	■	
	KP_017				■	■		■								■		■	
	KP_019					■				■		■	■	■					
	KP_026			■		■		■			■		■	■	■				
	KP_050			■		■		■					■	■	■		■	■	

	**KP_057**		■		■		■		■	■		■	■		■	
	**KP_059**		■		■		■		■	■		■	■			
	**KP_086**				■				■		■	■				
	**KP_095**		■		■		■		■	■			■		■	
Acinetobacter baumannii	**RAB_005**						■					■	■			
	**RAB_009**						■					■	■			
	**RAB_014**						■	■				■	■			
	**RAB_030**						■					■	■			
	**RAB_055**						■					■	■			

The black highlight ■ indicates the presence of the gene.
